# Development of practical pheromone lures for *Lygus hesperus* and *Lygus elisus* (Heteroptera: Miridae)

**DOI:** 10.1093/jee/toae266

**Published:** 2024-11-22

**Authors:** David R Hall, Jacqueline Serrano, Glenn Y Yokota, Diego J Nieto, Dudley I Farman, J Steven McElfresh, Alejandro I Del Pozo-Valdivia, Jocelyn G Millar, Kent M Daane

**Affiliations:** Natural Resources Institute, University of Greenwich, Chatham Maritime, Kent ME4 4TB, UK; Departments of Entomology and Chemistry, University of California, Riverside, CA 92521, USA; Department of Environmental Science, Policy and Management, University of California, Berkeley, CA 94720, USA; Department of Entomology, Driscoll’s Inc., Watsonville, CA 95076, USA; Natural Resources Institute, University of Greenwich, Chatham Maritime, Kent ME4 4TB, UK; Departments of Entomology and Chemistry, University of California, Riverside, CA 92521, USA; University of California Cooperative Extension, Salinas, CA 93901, USA; Departments of Entomology and Chemistry, University of California, Riverside, CA 92521, USA; Department of Environmental Science, Policy and Management, University of California, Berkeley, CA 94720, USA

**Keywords:** western tarnished plant bug, pale legume bug, monitoring, sticky cards, hexyl butyrate, (*E*)-2-hexenyl butyrate, (*E*)-4-oxo-2-hexenal

## Abstract

The mirid bugs *Lygus hesperus* (Knight) and *L. elisus* (van Duzee) are key pests of forage, fiber, and fruit crops. Our goals were to identify pheromone components produced by females of both species and to develop practical pheromone dispensers for use in monitoring these pests. Volatiles collected from virgin female *L. elisus* contained (*E*)-2-hexenyl butyrate (E2HB) as the major component with lesser amounts of hexyl butyrate (HB) and (*E*)-4-oxo-2-hexenal (E4OH) (ratio 117.2:100:17.1, respectively), whereas volatiles and solvent extracts from *L. hesperus* contained HB and E4OH as major components, with only small amounts of E2HB (100:23.6:3.4, respectively in volatiles). Dispensers fabricated from pipette tips released the components at ~10 µg/d in a ratio similar to the loading ratio. These lures were used to optimize the pheromone blends in field studies from 2012 to 2017. Blends of E2HB and E4OH attracted *L. elisus,* and a 100:60 blend was optimal. Blends of HB and E4OH attracted *L. hesperus*, and a 100:60 blend was adopted as a base blend. The additions of possible minor components such as (*Z*)-3-hexenyl butyrate, (*E*)-2-hexenal, or 1-hexanol did not improve the attraction of *L. hesperus*. In trials in alfalfa and strawberry, traps baited with blends of HB:E4OH (100:60) were equally or more effective for monitoring *L. hesperus* than sweep or vacuum samples, with pipette tip dispensers lasting 2–3 weeks under field conditions. The numbers of *L. hesperus* captured were lower than expected as compared with reports of pheromone trapping for other *Lygus* spp. Some possible reasons were investigated.

## Introduction

The western tarnished plant bug, *Lygus hesperus* (Knight), and the pale legume bug, *L. elisus* (van Duzee), are key pests of forage and fiber crops such as alfalfa and cotton (Barlow et al. 1999, Cooper and Spurgeon 2013), and fruit crops such as strawberries (Swezey et al. 2013, Zalom et al. 2014). On strawberries, feeding by both nymphal and adult *Lygus* spp. causes irregularly-shaped “cat-faced” berries (Allen and Gaede 1963). *Lygus* adults (and to some extent nymphs) disperse within and among different host plants as they search for new nutritional resources (Sivakoff et al. 2012, Swezey et al. 2013, Hagler et al. 2020, Nieto et al. 2023). Effective monitoring for *L. hesperus* throughout the season can better synchronize management efforts, such as insecticide applications or deployment of tractor-mounted vacuums, with the immigration of these pests from alternate hosts into crops. While a number of different sampling methods have been investigated for *Lygus* spp. (Blackmer and Cañas 2005, Villavaso 2005, Musser et al. 2009, George et al. 2023a), sweep nets are still commonly used to monitor population presence and density (Schotzko and Okeeffe 1986, Zalom et al. 1993). However, at low *Lygus* densities (e.g., during their initial reproductive period in spring), sweep nets are not effective for sampling these insects in most crop systems. Alternative sampling strategies have been explored, such as assessing the attraction of *L. hesperus* adults and nymphs to plant volatiles and visual cues (Blackmer et al. 2004, 2008, Williams et al. 2010). More sensitive tools, such as effective pheromone-baited traps that could detect the earliest movement of the insects into crops, would further advance the management of *Lygus* spp.

The use of insect pheromones as attractants in monitoring traps has revolutionized sampling methods for many of the major insect pests of agricultural crops (Witzgall et al. 2010). Pheromone-baited traps can provide a simple, effective, and highly selective method to determine the phenology and density of insect populations and inform pest management decisions. Traps and attractant baits are now available for many pest insect species from different insect orders, such as moths, beetles, and flies. However, to date, the discovery of attractant pheromones for true bug (heteropteran) species has lagged that of insects in other orders, in part because the identification of pheromones for these insects is complicated by the large amounts of volatile defensive chemicals that many true bugs produce (e.g., Ho and Millar 2002, Moreira and Millar 2005, Byers 2006). These defensive compounds are generally produced in much greater abundance than the pheromone components and can therefore obscure the latter during analyses.

Some of the principal aspects of intraspecific attraction of *Lygus* spp. have been previously examined. Attraction of male *L. hesperus* to virgin female conspecifics was reported by Strong et al. (1970), McLaughlin (1996), and Graham (1987, 1988). Ho and Millar (2002) later identified 17 compounds in headspace extracts and extracts of metathoracic glands, with hexyl butyrate (HB) and (*E*)-2-hexenyl butyrate (E2HB) being the major components. However, no qualitative differences were found among volatiles from female and male insects, and there were no qualitative differences in the antennal responses of females or males to conspecific extracts. Furthermore, in field bioassays, neither nymphs nor adults of either sex were attracted to any of the 120 possible binary combinations of 16 of the 17 compounds identified in the aeration extracts, excluding (*E*)-4-oxo-2-hexenal (E4OH). Byers et al. (2013) reexamined the female-produced sex pheromone of *L. hesperus,* as well as those of *L. lineolaris* (Palisot de Beauvois) (Heteroptera: Miridae) and *L. elisus*. These authors proposed that HB, E2HB, and E4OH were pheromone components of all 3 species, and that the correct relative ratios of these compounds were critical to achieve conspecific attraction and heterospecific avoidance. Accordingly, males of *L. hesperus* were found to be attracted to traps baited with a 3:0.3:2 blend of HB, E2HB, and E4OH, while males of *L. elisus* and *L. lineolaris* were attracted to a 1.2:3:2 blend of these 3 compounds (Byers et al. 2013). Fountain et al. (2014) similarly reported that the pheromone blends produced by 4 European mirid species contained HB, E2HB, and E4OH.

In the field experiments carried out by Byers et al. (2013), compounds were dispensed from open glass vials and had to be renewed daily, thus reducing the practicality for field monitoring. Fountain et al. (2014) described a dispenser containing these blends that released the compounds in similar blends to those loaded in the dispenser, with release ratios being stable for over 2 months under laboratory conditions. Thus, our objectives in the work described here were twofold: (i) reexamine the volatiles produced by female *L. hesperus*, with the goal of determining the blend of compounds that were both necessary and sufficient for the attraction of males; (ii) develop a practical dispenser for this synthetic pheromone blend that could be used in traps for long-term monitoring of *L. hesperus* males. During this work, we expanded our objectives to include the development of a pheromone lure for *L. elisus* based on the observed attraction of these males to test treatments deployed for *L. hesperus*.

## Materials and Methods

### Collection of Volatiles from *Lygus* spp.

Collections of volatiles and whole-body solvent extracts of *Lygus* spp. were made at the University of California Riverside (UCR) and the Natural Resources Institute, UK (NRI). At UCR, colonies of *L. hesperus* and *L. elisus* were maintained from specimens collected in alfalfa and native vegetation. Insects were reared in a bug dorm (BioQuip; Rancho Dominguez, CA, USA) maintained at 24 °C with 60%–75% relative humidity under long-day conditions (18:6 h L:D) to ensure that they remained reproductively active. Both colonies were reared on a diet of organically grown green beans, eggs of navel orangeworm, *Amyelois transitella* (Walker), and raw sunflower seeds (Ho and Millar 2002). Eggs were laid in green beans, which were removed every 2–3 days and transferred to ~1.9 L polyethylene cups with side screens. The resulting nymphs were held in these containers until they reached adulthood, using the diet described above. To obtain virgins for analyses, males and females were separated by sex immediately before adult eclosion, according to the presence or lack of a developing ovipositor, and were held individually in 20-ml vials with screen caps and a piece of green bean until the final molt. The resulting virgin adults were then used for the collection of headspace volatiles once they were sexually mature. Newly emerged *Lygus* adults were sexed and held in single-sex groups for 5 days before use to ensure that they were sexually mature.

To collect pheromone from *L. elisus*, sexually mature females were individually transferred to 250-ml glass aeration chambers with a Teflon lid and a green bean at least 12 h before aerations were performed. This was done to minimize disturbance and handling of the insects prior to the start of aerations. Charcoal-filtered air was pulled through the chambers using house vacuum with a flow rate of 250–300 ml/min. The volatiles produced by the bugs were collected for 3, 48-h periods over 6 consecutive days on activated-charcoal traps consisting of a ~0.5 cm bed of 100 mesh activated-charcoal held between glass wool plugs in a 4 mm ID disposable glass tube. Collectors were extracted with 0.5 ml of dichloromethane, and the extracts were stored at −20 °C until analysis. In addition, the metathoracic scent glands (MSG) containing defensive secretions were dissected out of sexually mature *L. hesperus* bugs, and the glands were extracted in hexane as described in Yang et al. (2015). The resulting extracts were then transferred to clean vials and held at −20 °C until analyzed.

For collection of volatiles at NRI, eggs of *L. hesperus* laid on green beans were sent by courier from Kearney Agricultural Research and Extension Center to the UK (under invertebrate license 24569/210645/5A). The insects were reared through to adults in a quarantine facility and fed on green beans under 12:12 h L:D conditions with temperatures of 25 and 20 °C, respectively, and 50% RH. Fifth instar nymphs were separated by sex and reared to adults in individual petri dishes (9 cm dia.) with a green bean. Volatiles were collected from undisturbed, individual, virgin female or male *L. hesperus* adults at 3–7 days old for 24 h periods under the same conditions as those used for rearing. The insects were housed in silanized glass vessels (12 cm × 4 cm) with a single green bean, and air was drawn into the vessel at 1 L/min through a filter (20 cm × 2 cm) containing activated charcoal (10–18 mesh) and out through a collection filter containing Porapak Q (200 mg; 50–80 mesh; Supelco, Gillingham, Dorset, UK) held between plugs of glass wool in a Pasteur pipette (4 mm inner dia.). The Porapak Q was initially purified by Soxhlet extraction with chloroform, followed by washing with dichloromethane before each use. Volatiles were eluted from the filter with dichloromethane (1 ml; Pesticide Residue Grade) for analysis. Whole-body extracts of virgin female and male *L. hesperus* adults were made by immersing individuals in diethyl ether (0.5 ml) for 10 min, removing the solvent, and washing with an additional 0.5 ml of diethyl ether.

### Analysis of Volatiles from *Lygus* spp.

At UCR, extracts of volatiles from *L. elisus* were analyzed on a HP 5890 Series II GC (Agilent, Santa Clara CA, USA) fitted with a DB-17 column (30 m × 0.25 mm ID, 0.25µm film; J&W Scientific, Folsom CA, USA). Injections were made in splitless mode at 250 °C, and the oven temperature program was 50 °C for 1 min, then 10 °C/min to 250 °C and held for 20 min. Authentic standards were also analyzed using the same GC parameters. The *L. hesperus* MSG extracts were analyzed by coupled gas chromatography-mass spectrometry (GC-MS) using an Agilent 6890N GC interfaced to a 5975 mass selective detector. The GC was fitted with a DB-5 column (J&W Scientific, 30 m × 0.25 mm, 0.25 µ film thickness), and injections were made in splitless mode with helium carrier gas. The GC oven was programmed from 40 °C for 1 min and increased at 10 °C/min to 280 °C, then held for 10 min. Injector and transfer line temperatures were 250 and 280 °C, respectively. Mass spectra were taken with electron impact ionization (70 eV), with a *m/z* range of 40–400. Compounds in the extracts were tentatively identified by matching their mass spectra with NIST14 database spectra. Identifications were confirmed by matching mass spectra and GC retention times of extracted compounds with those of authentic standards.

At NRI, decyl acetate (5 µg) was added to both the volatile collections and whole-body extracts as an internal standard. Samples were analyzed on a HP6850 GC (Agilent, Cheadle, UK) fitted with a fused-silica column (30 m × 0.32 mm i.d., 0.25 µ film thickness) coated with polar DBWax (Supelco), with helium carrier gas (2.4 ml/min), splitless injection (220 °C), and flame ionization detection (FID) (250 °C). The oven temperature was programmed from 50 °C for 2 min, then increased by 10 °C/min up to 250 °C, which was held for 5 min. The identity of compounds was confirmed by GC-MS on a CP-3800 GC coupled to a Saturn 2200 MS (Varian, now Agilent) using a DBWax column (30 m × 0.25 mm i.d., 0.25 µ film thickness; Supelco) with helium carrier gas (1 ml/min). The oven temperature was programmed from 40 °C for 2 min, then increased by 10 °C/min to 250 °C.

Samples were also analyzed by gas chromatography coupled to electroantennography (GC-EAG) at NRI using an HP6890 GC (Agilent) fitted with a flame ionization detector (FID) and DBWax and DB5 columns (30 m × 0.32 mm i.d., 0.25 µm film thickness; Supelco). Injections onto the DBWax column were in splitless mode (220 °C), carrier gas was helium (2.4 ml/min), and the oven temperature program was 50 °C for 2 min, then increased at 10 °C/min to 250 °C, and held 3 min. The effluents of the 2 columns were combined with a glass push-fit Y-tube connector (Agilent) connected to a second Y-tube connector with deactivated fused silica tubing (10 cm × 0.32 mm i.d.). One arm of this connector was attached with deactivated fused silica tubing (50 cm × 0.32 mm i.d.) to the FID (250 °C), while the other end was connected to an equal length of deactivated silica tubing passing through a heated transfer line (250 °C; Syntech, Hilversum, The Netherlands, now Kirchzarten, Germany) and into a glass tube (4 mm i.d.), through which air passed (500 ml/min) over the EAG preparation.

EAG preparations were made using glass microelectrodes filled with saline (0.1M KCl with 1% polyvinylpyrrolidone) that were attached to silver wire electrodes and held by integrated electrode holders, micromanipulators, and an amplifier (INR-2; Syntech). The base electrode was inserted into the back of the excised head of a male *L. hesperus*, and the recording electrode was brought into contact with the tip of one antenna. The FID and EAG signals were collected and analyzed with EZChrom software (Elite v3.0; Agilent Technologies).

### Lure Preparation and Measurement of Release Rates

Candidate pheromone blends were formulated in pipette tip dispensers, which were reported to provide sustained release of these compounds in a ratio similar to that loaded in the dispenser by Fountain et al. (2014). HB and E2HB were obtained from Sigma-Aldrich (now Merck, Gillingham, Dorset, UK) and were >99% pure. E4OH was prepared as described by Moreira and Millar (2005) and Fountain et al. (2014). The blends were formulated in sunflower oil with the major component at 10% w/v and variable amounts of the other 2 compounds, depending on the experiment. 4-Methyl-2,6-di-*tert*-butylphenol (BHT; 10% of major component) and Waxoline Black (10% of major component; Kadion, Barcelona, Spain) were added as an antioxidant and UV blocker, respectively. The blend (100 µl per lure) was then applied to a cigarette filter pushed into a polypropylene disposable pipette tip (1 ml; Fisher Scientific, Loughborough, UK). Loaded tips were sealed with a Teflon-lined crimp seal and wrapped in duct tape to exclude light, leaving the small end of the pipette open. Lures were packed in heat-sealed aluminum foil bags for shipping to the US and stored in a refrigerator (4 °C) before use.

In one experiment, lures consisting of black low-density polyethylene bulbs (25 mm height × 6 mm dia × 0.25 mm thick; Pherobio Technology Co. Ltd., Beijing, China) (Zhang et al. 2020) were tested. These were loaded with the pheromone blend in sunflower oil (100 µl) as described above and heat sealed.

Release rates from the dispensers were measured as described by Fountain et al. (2014). Briefly, dispensers were maintained in a laboratory fume hood at 20–22 °C. At intervals, volatiles were collected by drawing charcoal-filtered air (2 L/min) over single lures held in a glass vessel (10 cm × 3 cm dia), trapping volatiles on filters containing Porapak Q (200 mg; 50–80 mesh; Supelco) as described above. Trapped volatiles were eluted from the filters with dichloromethane (1 ml) and analyzed by GC-FID using the same conditions as for the extracts of volatiles from live insects, with an internal standard of decyl acetate (2 µg). Collections were carried out for 3 h at 20–22 °C. The means of 2 replicates were used for analysis.

### Field Testing of Pheromone Blends

Field tests to determine the most effective blends of the pheromone components for trapping *Lygus* spp. were carried out between 2012 and 2016. Initial trials in 2012 were conducted in a 1.5 ha alfalfa block, which provided a predictable population of *Lygus*, at the Kearney Agricultural Research and Extension Center (KARE) near Parlier, CA. A second such block of alfalfa was added for experiments at KARE from 2013 onwards.

Traps were inverted sticky delta traps (Biolure Delta from Suterra Inc., Bend, OR, or Pherocon VI Delta from Trécé, Inc., Adair, OK) attached horizontally to a wooden dowel to be held upright in the field. Pipette tip lures were fastened inside the traps (Supplementary Material Fig. S1). Traps were positioned approximately 25 m apart in a randomized block design with 4 replicates of each treatment and an unbaited control trap in each of the 1.5 ha fields. In 2012, traps were checked every 3–6 days, at which point all *Lygus* spp. were counted and removed. From 2013, traps were similarly checked every 1–3 days. Lures were replaced every 3–4 weeks. Details of the blends of synthetic compounds tested are given in the Results section. In experiments where catches with synthetic lures were compared with catches with virgin female *L. hesperus,* 5 virgin females were enclosed in a plastic vial with organdy-covered lids, fed with fresh alfalfa cuttings, and placed inside each sticky trap (where they were protected from sunlight).

Additionally, before each harvest, alfalfa was sampled using a sweep net (100 sweeps per plot) and the number and species of *Lygus* collected using sweep net samples were compared to trap counts. *Lygus* species identifications were based on Mueller et al. (2003).

### Field Trapping in Alfalfa and Strawberry

The attractiveness of the optimized pheromone blend (100:60 HB:E4OH) to *L. hesperus* males was examined in alfalfa at KARE in 2017 and commercial strawberry farms in Monterey and Santa Cruz Counties, California, in 2018–2020 and 2023. In 2017, 2 experiments were carried out to investigate why trap catches of *L. hesperus*, using the 100:60 HB:E4OH blend, had been relatively low compared with earlier trap captures of *L. elisus*, using the 100:60 E2HB:E4OH blend, despite the apparently much higher densities of the former species in alfalfa based on sweep net samples. In the first experiment, trap catches of *Lygus* spp. were compared from Pherocon VI Delta sticky trap liners that were either baited with pheromone lures or left unbaited (*N* = 4). Traps were checked every 1–4 days to remove *Lygus* and thereby reduce the possibility that captured males would give off an alarm pheromone. Periodically, sweep net samples were taken around the traps to see if more males were drawn to the vicinity of the baited traps as compared with the unbaited traps. The trial ran from 2 May to 9 June 2017, with the pheromone lures replaced on 22 May 2017.

In the same alfalfa block, we also tested whether the pheromones produced by *L. hesperus* females in the field competed or interfered with the lures, thereby reducing the number of adult males being attracted into the traps. Five treatments were used to vary the density of *Lygus* near the pheromone lure. After the alfalfa was mowed, raked, and baled, 5 small strips of tall alfalfa (or berm) were left with everything else mowed close to the ground and supporting few, if any, *Lygus*. The distance between each alfalfa strip was 30.5 m. The treatments placed in the alfalfa berm included traps baited with virgin females as a natural lure, the 100:60 HB:E4OH blend, and an unbaited control. Traps baited with the synthetic lure were placed 7.6 and 15.2 m away from the berm and the resident *Lygus* population. There were 4 replicates in a randomized block design, and the *Lygus* were collected every 2–3 days during the trial from 2 to 18 August 2017.

To investigate the value of the pheromone traps for monitoring *L. hesperus* on commercial strawberry ranches, trials were positioned along field edges in blocks ranging from 1 to 6 ha. Production practices, such as irrigation, fertilization, and pest management, were led by collaborating growers and were standardized across treatment blocks. All treatment blocks used the same proprietary strawberry variety during each sample year. Trials in 2018 and 2019 were conducted on a single ranch and included 3 and 4 replicates, respectively. Trials in 2020 and 2023 consisted of one treatment comparison per ranch and included 10 and 4 replicates, i.e., ranches, respectively. From 2018 to 2020, data were collected from April to June, which coincides with immigration of *Lygus* spp. into strawberries. In 2023, data collection began in May and extended into August. Each replicate consisted of an inverted delta sticky trap using Pherocon VI Delta liners that was either unbaited or baited with the 100:60 HB:E4OH blend. Traps were positioned 10 strawberry rows (i.e., 12 m) from a field edge and were separated by at least 30 m. *Lygus* spp. were collected from traps, and lures were replaced weekly, while sticky cards were replaced every 2–3 weeks. *Lygus* spp. were also sampled from strawberries using a hand-held vacuum made from a retrofitted Stihl SH 56 C-E Shredder Vacuum (Stihl Incorporated, Virginia Beach, VA). Each sample consisted of 50 suctions directed at strawberry flowers along a single row. Suction samples were collected weekly from strawberry rows near both the pheromone-baited trap and the control trap, at 4, 12, and 24 m (2020 only) from a field edge. Collected specimens were taken to the Driscoll’s Entomology Laboratory, where they were identified to species and counted.

### Data Analyses

Data are presented as means ± SEM. For comparison of different pheromone blends, means were determined for each trial period and treatment. Treatment impact was determined using a General Linear Model (GLM) function, with treatment means separated using Tukey’s HSD pairwise comparison (*P* < 0.05). In each block and field, treatment location was re-randomized after each trial period, and therefore replicates were assigned as the means from each block for each field and trial period. In each year, trial periods repeating the same treatment sets were considered separate replicates. Data were transformed to log(x + 1) or square root(x + 0.5) as needed to normalize the variance. All analyses were performed using Systat Software Inc. (version 13, San Jose, CA, USA). For field studies that examined the effectiveness of HB:4OH in alfalfa (2017) and strawberry (2018–2020, 2023), season-long treatment effects were compared using the GLM function in Systat, with adult male *L. hesperus* densities as the dependent variable and treatment and date as the independent categorical variables, with a treatment × date interaction term.

## Results

### Analysis of Volatiles from *Lygus* spp.

Each *Lygus* spp. produced different ratios of volatiles. Volatiles collected from single, undisturbed, mature virgin female *L. elisus* contained E2HB as the major component, with significant amounts of both HB and E4OH (Table 1). Analyses of solvent extracts of the metathoracic glands of virgin male or female *L. hesperus* showed HB as the major component, with significant amounts of E4OH, but only small amounts of E2HB (Table 1). Volatiles from single virgin female *L. hesperus* contained HB, E2HB, and E4OH in similar relative amounts as found in the metathoracic glands (Table 1 and Supplementary Material Fig. S2). However, these compounds were not detected in volatiles collected from single virgin male *L. hesperus.* Traces of (*Z*)-3-hexenyl butyrate (Z3HB) were also detected in gland extracts and volatiles from *L. hesperus*. In studies at NRI, there was no consistent pattern of production of compounds during successive light and dark periods, although mating activity of *L. hesperus* is reported to occur mainly during the morning hours (Brent 2010). For 10 individuals, production of HB was 0.8 ± 0.17 µg/12 h/insect during the light period and 0.4 ± 0.15 µg/12 h/insect during the dark period (Supplementary Material Fig. S3).

**Table 1.** Analyses of volatiles collected from single, undisturbed, mature virgin female *Lygus elisus* and *L. hesperus* and analyses of solvent extracts of virgin male or female *L. hesperus.* Results (mean ± SE) are expressed relative to HB = 100, and studies were conducted at UCR and NRI, UK

					Proportion relative to HB = 100 ± SE				
Species	Sex	Method	Lab	*N*	E26Ald^1^	6OH	Z3HB	E2HB	E4OH
*L. elisus*	F	Aeration	UCR	7	-	-	-	117.2 ± 9.6	17.1 ± 11.4
*L. hesperus*	F	Extract	UCR	8	1.3 ± 0.5	1.4 ± 0.2	0.25 ± 0.01	5.4 ± 0.8	8.4 ± 0.9
*L. hesperus*	M	Extract	NRI	5	-	0.9 ± 0.4	0.14 ± 0.01	1.9 ± 0.8	20.9 ± 0.8
*L. hesperus*	F	Extract	NRI	5	1.8 ± 0.7	0.7 ± 0.2	0.15 ± 0.02	2.7 ± 0.5	16.7 ± 2.8
*L. hesperus*	F	Aeration	NRI	10	4.1 ± 0.8	4.4 ± 0.6	0.14 ± 0.04	3.4 ± 0.3	23.6 ± 1.9

^1^E26Ald, (*E*)-2-hexenal; 6OH, 1-hexanol; Z3HB, (*Z*)-3-hexenyl butyrate; E2HB, (*E*)-2-hexenyl butyrate; E4OH, (*E*)-4-oxo-2-hexenal.

Small and variable amounts of 1-hexanol (6OH) and (*E*)-2-hexenal (E26Ald) were detected in volatile collections but not in gland extracts. It is not clear whether these were from the insects or from the beans used as food. While neither compound was detected in volatiles collected from intact beans or beans that were artificially wounded with a dissecting needle, they may have been induced by insect feeding.

In linked GC-EAD analyses of volatiles collected from virgin female *L. hesperus*, antennae of male *L. hesperus* responded to HB, E2HB, and E4OH. No responses were observed to the small amounts of 6OH, E2-6Ald, or Z3HB present in the extracts (Supplementary Material Fig. S4). An EAG response was, however, elicited by a larger sample of synthetic Z3HB.

### Measurement of Release Rates from Lures

**Fig. 1.**
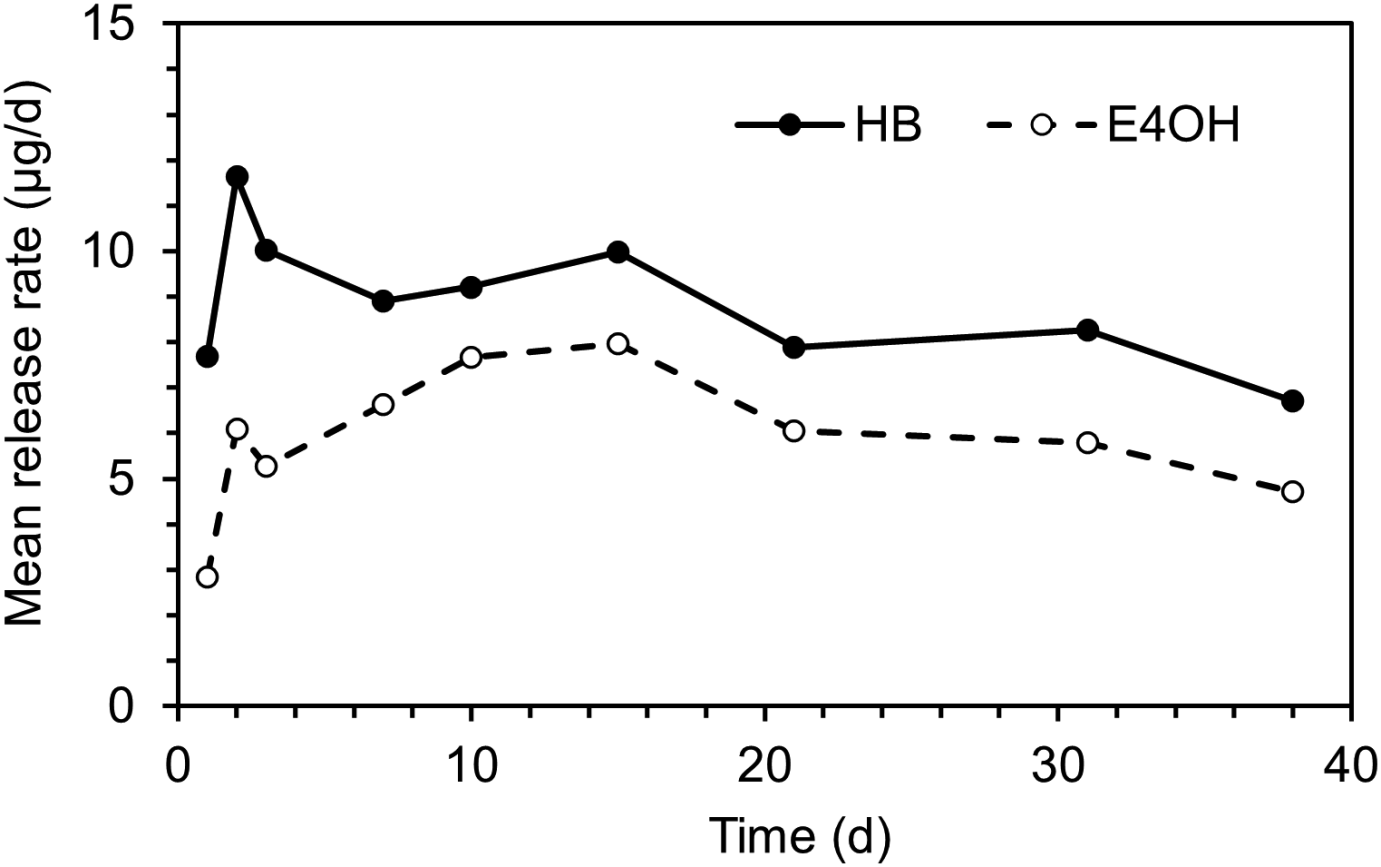
Release rates of HB and E4OH at 20–22 °C from a pipette tip dispenser loaded with 10 mg HB and 6 mg E4OH, measured by collection of volatiles; results are the mean of 2 replicates.

Release of HB and E4OH from a pipette tip dispenser loaded with 10 and 6 mg of the 2 components, respectively, showed that they were released in an ~10:7 ratio, with the major component released at ~10 µg/day (Fig. 1), which was a similar order of magnitude to that released by female *L. hesperus*. Release from the polyethylene bulb dispenser was twenty times more rapid (Supplementary Material Fig. S5), and the longevity correspondingly reduced.

### Field Testing of Pheromone Blends

In 2012, field trapping experiments compared catches in traps baited with blends of HB, E2HB, and E4OH based on previously reported ratios for several *Lygus* species, including *L. hesperus* by Byers (2006) (100:4:44) and Ho and Millar (2002) (100:4:2), *L. lineolaris* by Wardle et al. (2003) (100:27:8), and *L. rugulipennis* by Fountain et al. (2014) (100:3:20). Across 3 trial periods, catches of *Lygus* spp. in baited traps were significantly higher than those in unbaited traps, and traps baited with the 100:4:2 blend, containing a relatively low amount of E4OH, caught fewer *Lygus* spp. than those baited with the other blends (*F* = 11.48; *df* = 4, 15; *P* < 0.001) (Supplementary Material Fig. S6).

In 2013, the first trial compared catches in traps baited with all 3 and 2-component blends based on the most attractive blend of HB, E2HB, and E4OH tested in 2012 (100:27:8). Traps baited with a 25:7 blend of E2HB and E4OH caught significantly more *Lygus* than the other blends tested, which caught similar numbers as unbaited traps (*F* = 32.11, *df* = 4, 15, *P* < 0.001) (Supplementary Material Fig. S7A). In the second and third trial periods, this result was followed up by testing blends of E2HB and E4OH. Traps baited with blends of 100:30 and 100:100 E2HB:E4OH caught more *Lygus* spp. than 100:0, 100:50, 5:100, and 0:100 blends and the unbaited control (*F* = 8.28, *df* = 7, 120, *P* < 0.001) (Supplementary Material Fig. S7B).

During these trials, *L. hesperus* was the only *Lygus* species caught in sweep net samples from adjacent alfalfa, and it was assumed this was also the main species caught in the pheromone traps. However, in 2014, closer examination of the *Lygus* caught in the pheromone traps revealed that the predominant species was instead *L. elisus*. Thus, from 2014 onwards, all trapped insects were identified to species using characters described by Mueller et al. (2003).

**Fig. 2.**
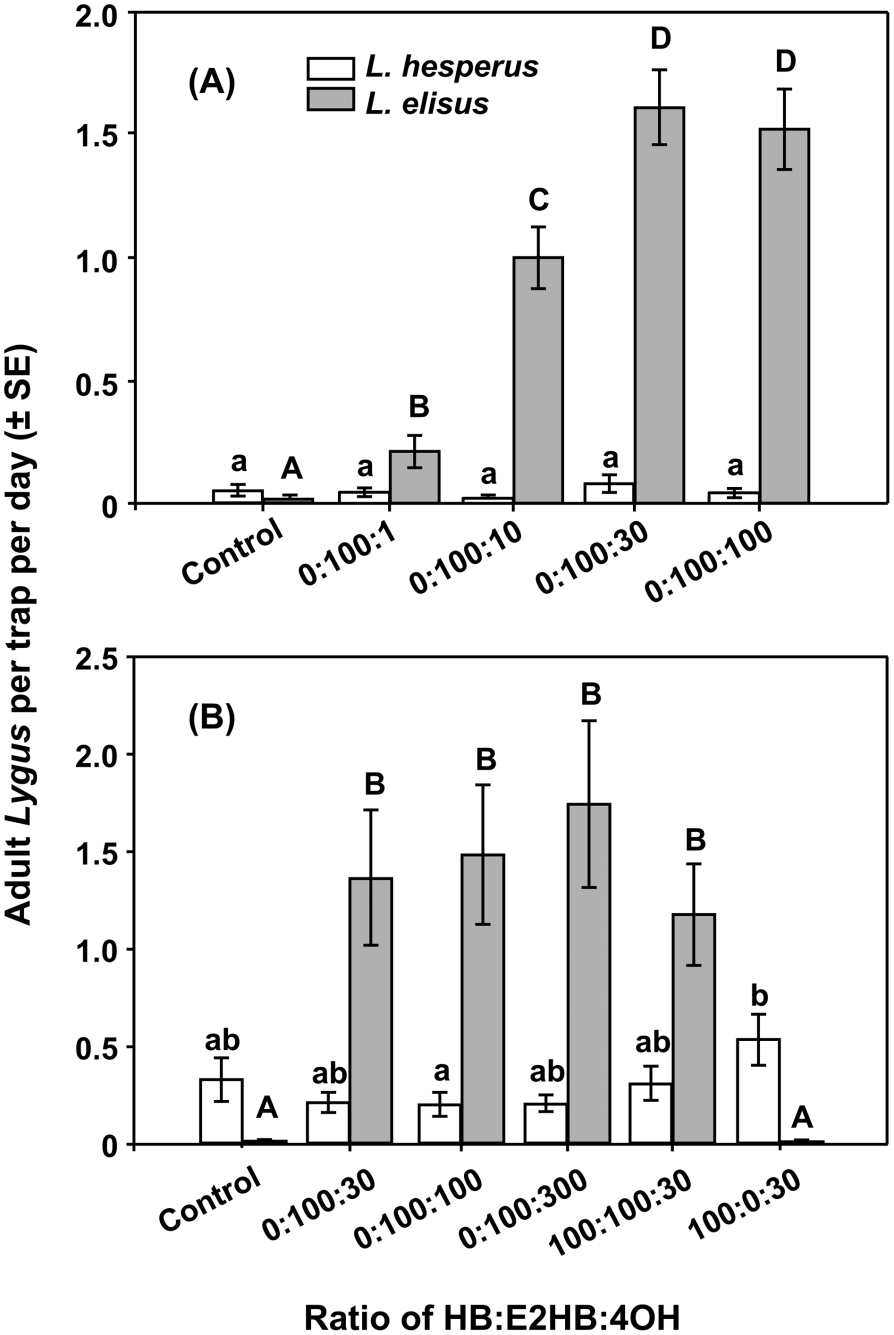
Trap captures of adult male *Lygus elisus* and *L. hesperus* (mean ± SE) in 2014 with lures containing blends of HB, E2HB, and E4OH in alfalfa fields during (A) trial periods 9–23 April and 6–28 May using blends of E2HB and E4OH and (B) trial periods 18 June–7 July and 14–25 July using blends of HB:E2HB:4OH. In each graph, means with different letters are significantly different (lower case for *L. hesperus* and upper case for *L. elisus*) (*P* < 0.05).

Two experiments were carried out across 4 trial periods during 2014, and across all treatments and sample dates, more *L. elisus* were captured than the target species *L. hesperus* (0.88 ± 0.6 vs. 0.05 ± 0.01 adult males/day, respectively) (*F* = 180.9; *df* = 1, 870; *P* < 0.001). During the first and second trial periods (9–23 April and 6–28 May 2014), the experiments of 2013 were partially repeated by comparing catches in traps baited with 100:1, 100:10, 100:30, and 100:100 blends of E2HB and E4OH only. None of the treatments tested attracted significant numbers of *L. hesperus* (*F* = 1.12; *df* = 4, 35; *P* = 0.36), but there was a treatment effect on catches of *L. elisus* (*F* = 33.98; *df* = 4, 35; *P* < 0.001), with blends of 100:30 and 100:100 E2HB:E4OH attracting more *L. elisus* than the 100:10 blend, which in turn was more attractive than the 100:1 blend (Fig. 2A). These results were similar to those in 2013 (Supplementary Material Fig. 6B), where it is assumed that catches were mainly *L. elisus.*

In the third and fourth trial periods during 2014 (18 June–7 July and 14–25 July 2014), blends of E2HB and E4OH (with higher proportions of E4OH) were tested, along with the effect of adding HB. Further increasing the proportion of E4OH relative to E2HB from 100:30 to 100:100 or 100:300 E2HB:E4OH did not increase catches of *L. elisus,* and a 100:60 blend of E2HB and E4OH was adopted as the standard blend for attraction of *L. elisus* in subsequent work. The addition of HB in a 100:100:30 blend of HB:E2HB:E4OH had no significant effect on catches of *L. elisus,* while omission of E2HB eliminated catches of *L. elisus* (*F* = 6.05; *df* = 5, 42; *P* < 0.001) (Fig. 2B). None of the treatments were significantly attractive to *L. hesperus* (*F* = 1.308; *df* = 5, 42; *P* = 0.28) (Fig. 2B). There was some indication that the addition of HB to the blend of E2HB and E4OH (100:100:30) and removal of E2HB from this blend (100:0:30) made it more attractive to *L. hesperus* (Fig. 2B), although the differences were not significant.

**Fig. 3.**
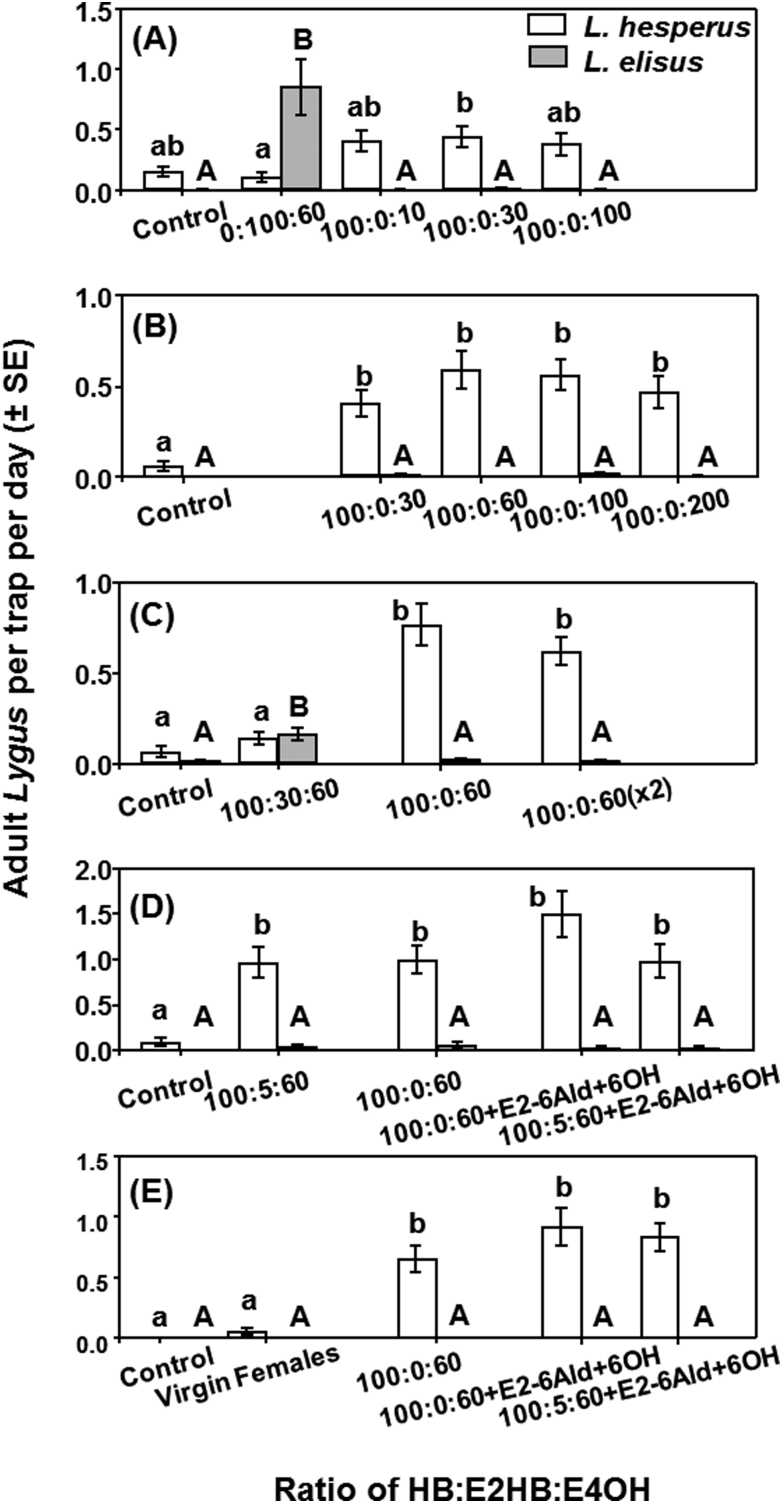
Trap captures of adult male *Lygus elisus* and *L. hesperus* (mean ± SE) in 2015 with lures containing blends of HB, E2HB, and E4OH in alfalfa fields during (A) trial period 1 (25 March–13 April), (B) trial period 2 (5–24 June), (C) trial period 3 (1–22 July), (D) trial period 4 (10–31 August) that added 5% (*E*)-2-hexenal (E2-6Ald) and 5% 1-hexanol (6OH) to the blend and (E) trial period 5 (23 September–14 October) that added virgin female lures as a second control. In each graph, means with different letters are significantly different (lower case for *L. hesperus* and upper case for *L. elisus*) (*P* < 0.05).

Based on the results from the trapping studies in 2014 and the data from the analyses of bug-produced volatiles, a series of 5 experiments was carried out in 2015, focusing on binary blends of HB and E4OH as potential attractants for male *L. hesperus*. Across all treatments (except the control) and trial periods, more *L. hesperus* (0.63 ± 0.03 adult males per day) than *L. elisus* (0.018 ± 0.003) were trapped (Fig. 3, *F* = 25.06; *df* = 1, 859; *P* < 0.001). During March–April, catches were compared in traps baited with 100:10, 100:30, and 100:100 blends of HB and E4OH, as well as those baited with the standard *L. elisus* lure containing E2HB and E4OH in a 100:60 ratio. There was a treatment effect on *L. hesperus* catches (*F* = 2.91; *df *= 4, 35; *P* = 0.036), with traps baited with the 100:30 HB:E4OH blend capturing more *L. hesperus* than those baited with the 100:60 blend of E2HB and E4OH (Fig. 3A). More *L. elisus* were captured in traps baited with this E2HB:4OH blend than in all other treatments (*F* = 9.19; *df* = 4, 35; *P* < 0.001), which essentially caught no *L. elisus* (Fig. 3A). In sweep net sampling carried out in April, June, July, August, and September, far more *L. hesperus* was detected in alfalfa (32.6 ± 4.1 males per 100 sweeps) than *L. elisus* (2.0 ± 0.3 males per 100 sweeps) (*F* = 54.53; *df* = 1, 90; *P* < 0.001), as was the case in previous seasons. It was also noted that catches of *L. hesperus* in unbaited traps were generally higher than catches of *L. elisus* (e.g., Figs. 2 and 3), further suggesting populations of the former were higher than those of the latter.

In the second trial carried out in June 2015, the range of blends of HB and E4OH was extended to compare catches in traps baited with 100:30, 100:60, 100:100, and 100:200 blends. More *L. hesperus* were captured in traps baited with the HB:E4OH blends than the control, but there was no difference among blend ratios (*F* = 11.59; *df* = 4, 35; *P* < 0.001) (Fig. 3B). Captures of *L. elisus* were uniformly very low with all the tested blends (*F* = 1.93; *df* = 4, 35; *P* = 0.13). In the third trial in July 2015, the effect of doubling the release rate of lures containing the 100:60 bend of HB and E4OH was investigated by baiting traps with 2 pipette tip lures. The effect of adding E2HB to the 100:60 HB:E4OH blend was also tested in a 3-component 100:30:60 blend of HB, E2HB, and E4OH. Traps baited with lures containing HB and E4OH caught more *L. hesperus* than those baited with the 3-component blend or the unbaited control, but doubling the release rate did not affect catches (*F* = 18.98; *df *= 3, 28; *P* < 0.001) (Fig. 3C). As the only blend containing E2HB, the 3-component blend attracted more *L. elisus* than all other treatments (*F* = 13.83; *df* = 3, 28; *P* < 0.001) (Fig. 3C).

In 2 further trials carried out in 2015, the effects of adding 5% of potential minor pheromone components detected in the analytical studies to the 100:60 blend of HB and E4OH were examined. These included E2HB, E2-6Ald, and 6OH. In the August trial, traps baited with all the test blends caught more *L. hesperus* than the unbaited control traps (*F* = 10.40; *df* = 4, 35; *P* < 0.001), but the addition of minor components did not affect catches (Fig. 3D). Catches of *L. elisus* were uniformly very low (*F* = 0.60; *df* = 4, 35; *P* = 0.67) (Fig. 3D). In the last trial period (September–October), traps baited with caged virgin female *L. hesperus* were included, but these had similar catches as the unbaited control traps and significantly lower captures than traps baited with the 100:60 blend of HB and E4OH with or without the minor components (*F* = 37.10; *df* = 3, 12; *P* < 0.001) (Fig. 3E). The females used as lures were under considerable stress, and although water and alfalfa cuttings were provided, many of the females were dead after 2–3 days in the trap.

**Fig. 4.**
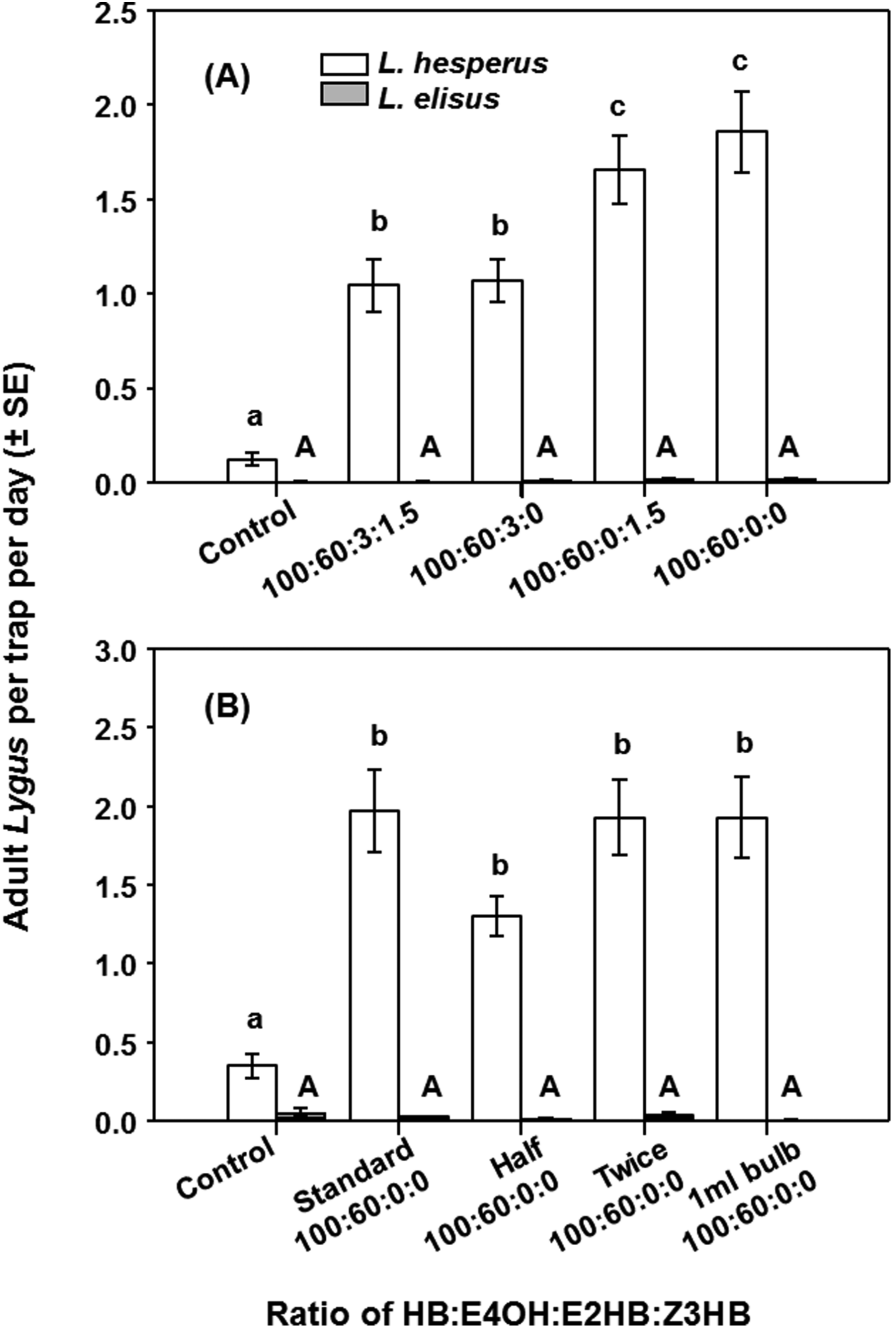
Trap captures of adult male *Lygus elisus* and *L. hesperus* (mean ± SE) in 2016 in (A) trial period 1 (18 April–16 May) with lures containing blends of HB, E4OH, E2HB, and Z3HB in a 100:60:3:1.5 ratio, respectively, or subsets thereof in alfalfa fields, and in (B) trial period 2 (27 June–10 August) that used a 100:60 HB:E4OH blend deployed at a standard, half, and double release rate as well as a 1 ml bulb release device. In each graph, means with different letters are significantly different (lower case for *L. hesperus* and upper case for *L. elisus*) (*P* < 0.05).

**Fig. 5.**
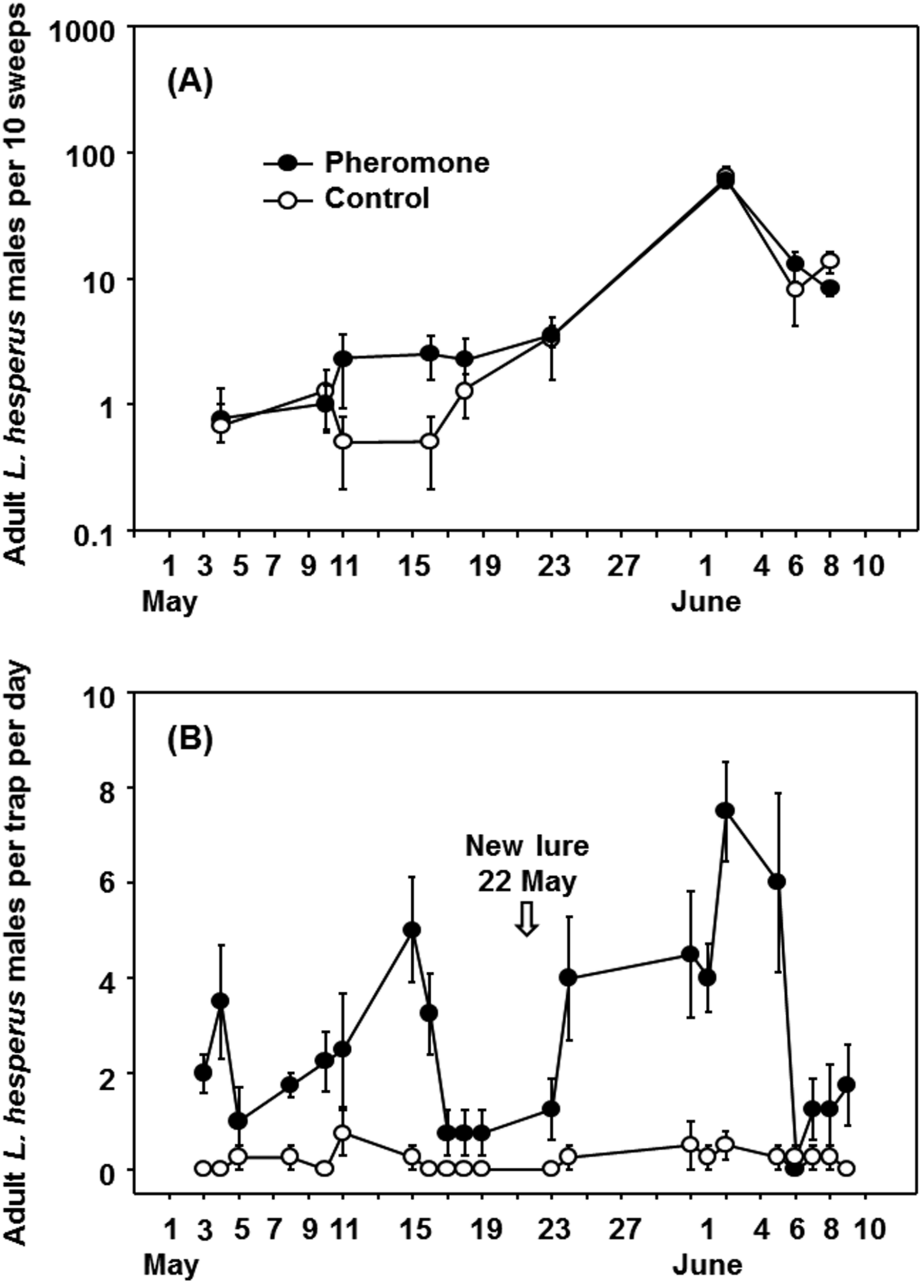
The number of adult *Lygus hesperus* (mean ± SE) captured in 2017 studies in alfalfa by (A) 10 sweep samples each on all 4 sides of baited and unbaited traps and (B) adult male *L. hesperus* trap captures per day (mean ± SE) over the same period using lures containing a 100:60 blend of HB and E4OH.

Experiments during 2016 further examined the effects of adding possible minor pheromone components and of adjusting pheromone release rates. In the first (18 April–2 May) and second (2–16 May) trial periods, catches were compared in traps baited with the 100:60 blend of HB and E4OH with and without E2HB and a potential minor component identified in volatiles from female *L. hesperus*, Z3HB (Table 1), at 3% and 1.5% of the HB, respectively. All baited traps captured more *L. hesperus* than the unbaited control traps, and the data suggest that addition of E2HB decreased attraction, whereas addition of Z3HB had no effect (*F* = 20.56; *df* = 4, 75; *P* < 0.001) (Fig. 5A). Trap captures of *L. elisus* were uniformly low, and no treatment was significantly different than the control (*F* = 0.82; *df* = 4, 75; *P* = 0.52) (Fig. 4A).

In the third (27 June–13 July 2016) and fourth (22 July–10 August 2016) trial periods, catches in traps baited with pipette tips containing the standard amount of pheromone were compared with those baited with pipette tips containing half or double the amount of pheromone, which were shown to release the pheromone blend at half or double the rate from the standard lure (data not shown) and the bulb dispensers with very high release rate. Traps baited with all the pheromone blends captured more *L. hesperus* than the unbaited control traps, with no difference among the half, standard, double dose, or the high release rate bulb dispenser (*F* = 11.21; *df* = 4, 75; *P* < 0.001) (Fig. 4B). There was no treatment effect on *L. elisus* captures (*F* = 1.30; *df* = 4, 75; *P* = 0.280) (Fig. 4B).

### Field Trapping in Alfalfa and Strawberry

In 2017, an experiment was carried out to investigate whether male *L. hesperus* were being attracted to the vicinity of traps but not actually entering the traps, perhaps because of the trap structure itself or an alarm pheromone emitted from trapped *Lygus*. There was no overall difference recorded from sweep net samples in alfalfa taken around traps baited with the 100:60 blend of HB and E4OH relative to samples taken around unbaited traps (*F* = 0.13; *df *= 1, 76; *P* = 0.72), suggesting this was not the case (Fig. 5A). However, the pheromone-baited traps captured more *L. hesperus* than unbaited traps over the sampled period (*F* = 79.13; *df* = 1, 166; *P* < 0.001) (Fig. 5B), although there was a clear decline in trap catches about 2 weeks after each new lure deployment (Fig. 5B). In terms of practical monitoring, the pheromone traps caught several *L. hesperus* per day early in the season, whereas sweep samples often captured fewer than one per 10 sweeps (Fig. 5A). However, numbers in sweep net samples increased significantly later in the season (note log scale in Fig. 5A) without a corresponding increase in catches in the pheromone-baited traps (Fig. 5B).

**Fig. 6.**
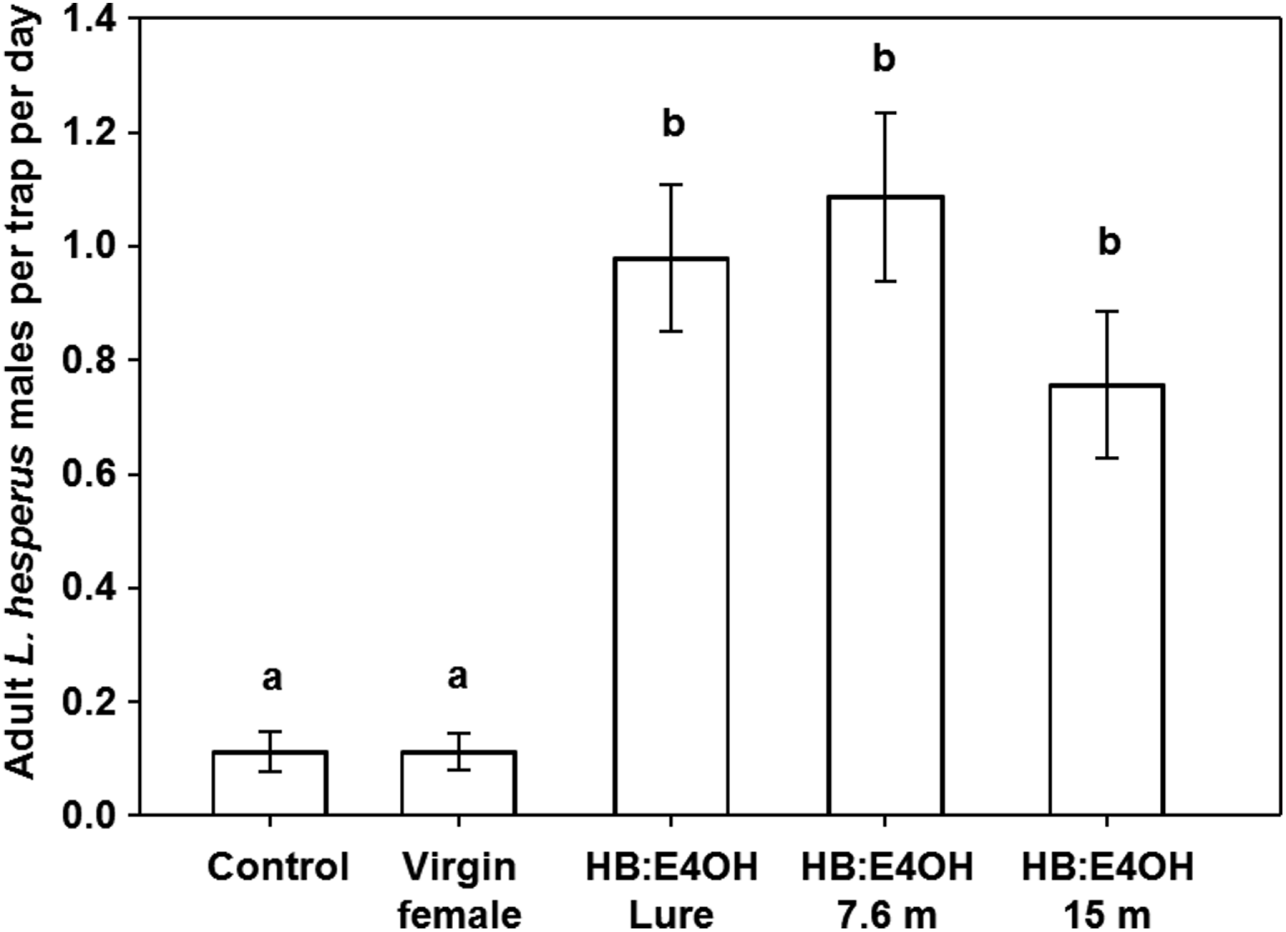
Mean catches of *Lygus hesperus* males in 2017 in traps in alfalfa (unbaited, baited with 4 virgin female *L. hesperus* or with a lure containing a 100:60 blend of HB and E4OH) compared with catches in traps baited with the pheromone lure at 7.6 and 15.2 m away from the alfalfa (*N* = 4; 2–18 August 2017); means with different letters are significantly different, *P* < 0.05).

In 2017, in an experiment to determine the effect of *L. hesperus* densities on catches in baited traps, pheromone traps containing a 100:60 blend of HB and E4OH were placed in a strip (berm) of alfalfa with a high density of *L. hesperus*, as well as at 7.6 and 15.2 m away from the berm where densities were assumed to be lower. Catches in pheromone-baited traps away from the berm were not significantly different from those caught on the berm (Fig. 6). However, more male *L. hesperus* were caught in traps baited with the synthetic pheromone than those in traps baited with conspecific virgin females (*F* = 31.35; *df* = 4, 20; *P* < 0.001). As noted above, the females used as lures did not survive well in these experiments.

**Fig. 7.**
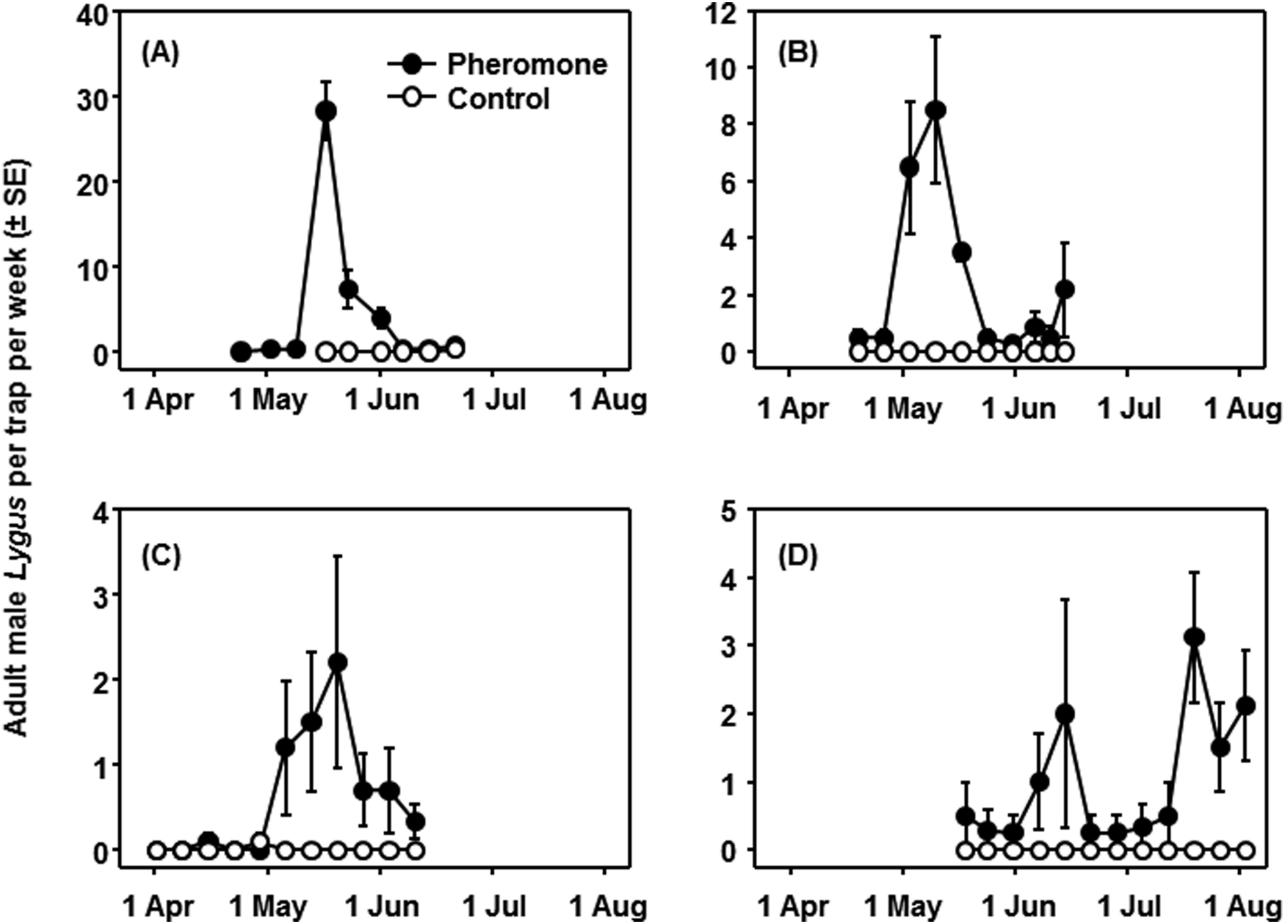
Mean number (± SE) of *Lygus hesperus* males captured on sticky traps with and without a pheromone lure on strawberry ranches in the Monterey Bay growing region in (A) 2018, (B) 2019, (C) 2020, and (D) 2023.

In further trials in strawberry fields, traps baited with the standard 2-component blend released from pipette tip dispensers captured more *L. hesperus* adult males than control traps in 2018 (*F* = 14.42; *df* = 1, 35; *P* < 0.001; Fig. 7A), 2019 (*F* = 22.66; *df* = 1, 69; *P* < 0.001; Fig. 7B), 2020 (*F* = 12.27; *df *= 1, 187; *P* < 0.001; Fig. 7C), and 2023 (*F* = 16.01; *df* = 1, 92; *P* < 0.001; Fig. 7D). Peak *L. hesperus* trap captures largely occurred during mid- to late-May (e.g., 2018, 2019, and 2020), but when traps were deployed later in the season in 2023, there were small peaks in June and late July as well. Also important is that over all seasons and sample dates, the pheromone traps captured more *L. hesperus* (1.60 ± 0.26 males per trap per week) than the control traps (0.010 ± 0.007) and significantly more than the standard vacuum samples from either near the pheromone traps (0.077 ± 0. 019 adults per sample) or near control traps (0.067 ± 0.014) (*F* = 46.79; *df* = 3, 1185; *P* < 0.001).

## Discussion

The laboratory and field work described here provide further confirmation that at least several species of *Lygus* use blends of hexyl butyrate (HB), (*E*)-2-hexenyl butyrate (E2HB), and (*E*)-4-oxo-2-hexenal (E4OH) in their female-produced sex pheromones (Byers et al. 2013, Fountain et al. 2014, Yang et al. 2015, Zhang et al. 2020, 2021). For *L. hesperus*, analyses of volatiles released by individual, undisturbed virgin females showed HB, E2HB, and E4OH in a 100:4.1:23.6 ratio, respectively, i.e., with HB as the major component. These compounds were not released by individual, undisturbed virgin males. Similar blends were observed in extracts of the metathoracic glands of both females and males, and these are in agreement with those reported by Byers (2006). All 3 compounds elicited electroantennographic responses from the antennae of male *L. hesperus*.

While our primary goal was the identification of attractive blends for *L. hesperus*, we also collected and analyzed volatiles from individual, virgin female *L. elisus*. These also produced blends of HB: E2HB: E4OH, in a 100:117.2:17.1 ratio, respectively, i.e., with E2HB as major component.

The various possible pheromone components were formulated as 10% blends in sunflower oil in pipette tip dispensers, with 10 mg of the major component, to give lures with release rates of similar orders of magnitude to those from virgin female *L. hesperus*, avoiding the repellent effects caused by high release rates (Wardle et al. 2003, Byers 2006, Byers et al. 2013). In laboratory studies, these dispensers released a similar blend to that loaded in the dispenser for at least 38 days under laboratory conditions and for 15 days under the higher temperatures experienced in the field. These results confirmed the versatility and longevity of this type of dispenser, as has been previously demonstrated for European species of *Lygus* (Fountain et al. 2014) and *L. lineolaris* (George et al. 2023b).

In our initial field experiments, *L. elisus* rather than the main target *L. hesperus* was the first species that was caught in significant numbers, and so lures for this species were optimized first. Lures containing E2HB and E4OH in 100:30, 100:100, or 100:300 blends attracted similar numbers of male *L. elisus*. Reducing the proportion of E4OH (e.g., 100:10 or 100:1 blends of the 2 compounds, respectively) resulted in lower catches. The addition of HB to produce a 100:100:30 ratio to simulate the blend observed in volatiles from the female insects did not increase or decrease catches relative to catches with the binary blend of E2HB and E4OH. These results are consistent with those of Byers et al. (2013), who showed that traps baited with a blend of HB, E2HB, and E4OH in a 1.2:3:2 ratio caught similar numbers of male *L. elisus* as those baited with the corresponding binary blend of E2HB and E4OH only, while removal of E2HB or E4OH from the blend caused it to become unattractive.

In contrast, the key components of the *L. hesperus* blend proved to be HB and E4OH. Thus, traps baited with binary blends of HB and E4OH in ratios of 100:30, 100:60, 100:100, and 100:200 caught similar numbers of male *L. hesperus* and significantly more males than traps baited with virgin females. Reducing the proportion of E4OH to 100:10 HB + E4OH reduced catches, as did the addition of E2HB. Analyses of volatiles collected from female *L. hesperus* showed traces of (*E*)-2-hexenal, 1-hexanol, and (*Z*)-3-hexenyl butyrate, but addition of these to a 100:60 blend of HB and E4OH did not affect catches. Doubling or halving the release rate of pheromone did not affect catches. These results are similar to those of Byers et al. (2013), who showed that a binary 3:2 blend of HB and E4OH was as attractive to male *L. hesperus* as a ternary 3:3:2 blend of HB, E2HB, and E4OH, although those authors did not evaluate blends closer to the blend released by the females. Removal of HB or E4OH from the ternary blend caused loss of attractiveness to male *L. hesperus*. Thus a 100:60 binary blend of E2HB and E4OH is proposed as a lure for male *L. elisus*, and a 100:60 blend of HB and E4OH as a lure for male *L. hesperus*.

The pheromone traps for *L. elisus* caught significant numbers of adult males even when this species was not commonly found in sweep net samples. The sweep sampling captured primarily *L. hesperus*, especially early in the season, but during experiments to optimize the pheromone blend for the latter species, numbers caught in the pheromone-baited traps were lower than those for *L. elisus.* This was unexpected in view of the greater numbers of *L. hesperus* shown to be present, and, as described above, some possible causes of this discrepancy were ruled out, such as the role of possible minor components in blends.

During the development of a pheromone lure for *L. hesperus*, it was somewhat surprising that trap catch rates remained so low relative to other *Lygus* spp. (e.g., George et al. 2023b). This may be attributed to several factors. For instance, many Hemiptera use substrate-borne vibrations for intraspecific communication (Cocroft and Rodriguez 2005), including *Lygocoris pabulinus* (L.) (Heteroptera: Miridae) (Drijfhout and Groot 2001), and incorporation of vibratory signals into pheromone-baited traps has been shown to increase catches of brown marmorated stinkbug, *Halyomorpha halys* (Stål) (Hemiptera; Pentatomidae) (Zapponi et al. 2023). Laboratory observations indicated that *L. hesperus* emits substrate-borne vibrations during mating (KMD, unpublished observations). Preliminary experiments were carried out in which catches in traps baited with virgin female *L. hesperus* and a synthetic lure were compared with those in traps baited with the synthetic lure alone, but no differences were observed (data not shown). However, the females did not survive well and may not have called, and/or they may not have produced vibrational signals. More detailed experiments, for example, recording vibrational signals from traps baited with virgin females, may be required to help resolve these uncertainties.

Another possibility for the relatively low trap catches could be the deterrent effects of emission of an alarm pheromone by *L. hesperus* males captured in the traps. Thus, an experiment was carried out in which the dying and dead males were removed from traps every day (data not shown); however, the capture rates were similar to those from the previous year’s trials. This suggested that dead or dying male *Lygus* in the traps were not emitting a volatile that dramatically altered the results, and this avenue of study was not further explored.

Our data did suggest that competition between the pheromone traps and resident females affected trap catches in 2 ways. Thus, we caught high numbers of *L. elisus*, relative to *L. hesperus*, when populations of the former were barely detectable by sweep net sampling, resulting in minimal competition from female *L. elisus* to the pheromone-baited traps. Furthermore, in the 2017 experiment trapping *L. hesperus* at different distances from a source of infestation, catches in the pheromone traps were similar at all distances away from the berm as in the berm. This could be interpreted in terms of competition from pheromone produced by the resident population in the berm reducing relative catches in the traps there and/or that the traps outside the berm were pulling in insects from their location in the berm. Whatever the reason, these results indicate that the current trap and lure for *L. hesperus* may be more effective earlier in the season and less effective later in the season.

It is also possible that improvements in trap design could increase catches of both *Lygus* species, but this was only briefly investigated here. In sweep net samples, there was no difference between numbers of *L. hesperus* around pheromone traps and those away from the traps. If insects were being attracted to the pheromone traps but then not entering the traps or not being caught, then we would have expected a significant buildup of insects around baited traps, although sampling may not have been done at the optimum time. In experiments with *L. rugulipennis* in Europe (Fountain et al. 2017), catches of males in cross vane bucket traps were up to 10-fold greater than in delta sticky traps. It was also observed that *L. rugulipennis* were able to free themselves from sticky traps coated with “wet” polybutene glue, and more *Lygus* spp. were caught in commercial “dry” glue traps (Fountain et al. 2017). Standard bucket “Unitraps” were also used effectively for trapping *L. pratensis* (L) (Heteroptera: Miridae) (Zhang et al. 2021). With *L. lineolaris*, trap color was also a factor, with more adult males caught on pheromone-baited, commercial red sticky cards than on similar blue, yellow, or white cards, and more were caught on cards coated with dry glues than on cards with wet glues (George et al. 2023a, 2023b). Thus, tests of different trap designs with *L. hesperus* may prove fruitful.

In summary, more than a decade of field testing of lure blends has yielded a reliable lure for *L. elisus*. Our results with *L. hesperus* are more equivocal: with our optimized 2-component blend, trap catches are consistently better than controls, but trap catches do not appear to scale with population density. Thus, the current trap and lure will be useful for detection of the onset of immigration of *L. hesperus* into crops but may not yet be sufficiently reliable for assessing population densities.

## Supplementary data

Supplementary data are available at *Journal of Economic Entomology* online.

toae266_suppl_Supplementary_Material

## Data Availability

All field trial raw data and other data not explicitly given in the manuscript, tables, or figures are available upon request from the authors.

## References

[CIT0001] Allen WW , Gaede SE. 1963. Relationship of *Lygus* bugs and thrips to fruit deformity in strawberries. J. Econ. Entomol. 56:823–825. https://doi.org/10.1093/jee/56.6.823

[CIT0002] Barlow VM , Godfrey LD, Norris RF. 1999. Population dynamics of *Lygus hesperus* (Heteroptera: Miridae) on selected weeds in comparison with alfalfa. J. Econ. Entomol. 92:846–852. https://doi.org/10.1093/jee/92.4.846

[CIT0003] Blackmer JL , Cañas LA. 2005. Visual cues enhance the response of *Lygus hesperus* (Heteroptera: Miridae) to volatiles from host plants. Environ. Entomol. 34:1524–1533. https://doi.org/10.1603/0046-225x-34.6.1524

[CIT0005] Blackmer JL , Rodriguez-Saona C, Byers JA, et al 2004. Behavioral response of *Lygus hesperus* to conspecifics and headspace volatiles of alfalfa in a Y-tube olfactometer. J. Chem. Ecol. 30:1547–1564. https://doi.org/10.1023/b:joec.0000042067.27698.30 15537158

[CIT0004] Blackmer JL , Byers JA, Rodriguez-Saona C. 2008. Evaluation of color traps for monitoring *Lygus* spp.: design, placement, height, time of day, and non-target effects. Crop Prot. 27:171–181. https://doi.org/10.1016/j.cropro.2007.05.003

[CIT0006] Brent CS. 2010. Reproduction of the western tarnished plant bug, *Lygus hesperus*, in relation to age, gonadal activity and mating status. J. Insect Physiol. 56:28–34. https://doi.org/10.1016/j.jinsphys.2009.08.021 19729015

[CIT0007] Byers JA. 2006. Production and predator-induced release of volatile chemicals by the plant bug *Lygus hesperus*. J. Chem. Ecol. 32:2205–2218. https://doi.org/10.1007/s10886-006-9140-x 17001534

[CIT0008] Byers JA , Fefer D, Levi-Zada A. 2013. Sex pheromone component ratios and mating isolation among three *Lygus* plant bug species of North America. Naturwissenschaften 100:1115–1123. https://doi.org/10.1007/s00114-013-1113-7 24233237

[CIT0009] Cocroft RB , Rodriguez RL. 2005. The behavioral ecology of insect vibrational communication. Bioscience 55:323–334.

[CIT0010] Cooper WR , Spurgeon DW. 2013. Feeding injury to cotton caused by *Lygus hesperus* (Hemiptera: Miridae) nymphs and prereproductive adults. Environ. Entomol. 42:967–972. https://doi.org/10.1603/EN13052 24331607

[CIT0011] Drijfhout FP , Groot AT. 2001. Close-range attraction in *Lygocoris pabulinus* (L.). J. Chem. Ecol. 2001:1133–1149. 10.1023/a:1010311928256 11504019

[CIT0012] Fountain MT , Cross JV, Jåstad G, et al 2014. Further studies on sex pheromones of female *Lygus* and related bugs (Heteroptera: Miridae): development of effective lures and investigation of species-specificity. J. Chem. Ecol. 40:71–83. 24390623 10.1007/s10886-013-0375-z

[CIT0013] Fountain MT , Baroffio C, Borg-Karlson A-K, et al 2017. Design and deployment of semiochemical traps for capturing *Anthonomus rubi* Herbst (Coleoptera: Curculionidae) and *Lygus rugulipennis* Poppius (Hetereoptera: Miridae) in soft fruit crops. Crop Prot. 99:1–9. https://doi.org/10.1016/j.cropro.2017.05.001

[CIT0014] George J , Glover JP, Reddy GVP, et al 2023a. Early season monitoring of tarnished plant bug, *Lygus lineolaris*, in wild hosts using pheromone traps. Insects 14:805. https://doi.org/10.3390/insects14100805 37887817 PMC10607691

[CIT0015] George JG , Reddy GVP, Little N, et al 2023b. Combining visual cues and pheromone blends for monitoring and management of the tarnished plant bug *Lygus lineolaris* (Hemiptera: Miridae). Pest Manag. Sci. 79:2163–2171. https://doi.org/10.1002/ps.7395 36730090

[CIT0016] Graham HM. 1987. Attraction of *Lygus* spp. (Hemiptera: Miridae) males by conspecific and congeneric females. Southwestern Entomol. 12:147–155.

[CIT0017] Graham HM. 1988. Sexual attraction of *Lygus hesperus* Knight (Heteroptera: Miridae). Southwestern Entomol. 13:31–37.

[CIT0018] Hagler JR , Nieto DJ, Machtley SA, et al 2020. Predator demographics and dispersal in alfalfa trap-cropped strawberry. Entomol. Exp.Appl. 168:53–58.

[CIT0019] Ho HY , Millar JG. 2002. Identification, electroantennogram screening, and field bioassays of volatile chemicals from *Lygus hesperus* Knight (Heteroptera: Miridae). Zool. Stud. 41:311–320.

[CIT0020] McLaughlin JR. 1996. Population monitoring of *Lygus hesperus* with female baited traps. Proc Beltwide Cotton Production Research Conf: 733–734.

[CIT0021] Moreira JA , Millar JG. 2005. Short and simple syntheses of 4-oxo-(*E*)-2-hexenal and homologs: pheromone components and defensive compounds of Hemiptera. J. Chem. Ecol. 31:965–968. https://doi.org/10.1007/s10886-004-1978-1 16124263

[CIT0022] Mueller SC , Summers CG, Goodell PB. 2003. A field key to the most common *Lygus* species found in agronomic crops of the central San Joaquin Valley of California. Davis (CA): Univ of California Agriculture and Natural Resources Publications 8104 [accessed 2012 Jan]. http://cottoninfo.ucdavis.edu/IMAGES/lygus8104.pdf.

[CIT0023] Musser FR , Catchot AL, Stewart SD, et al 2009. Tarnished plant bug (Hemiptera: Miridae) thresholds and sampling comparisons for flowering cotton in the midsouthern United States. J. Econ. Entomol. 102:1827–1836. https://doi.org/10.1603/029.102.0513 19886447

[CIT0024] Nieto DJ , Hagler JR, Swezey SL, et al 2023. Immigration of *Lygus* spp. (Hemiptera: Miridae) and predaceous natural enemies to trap-cropped organic strawberry. Environ. Entomol. 52:824–831. https://doi.org/10.1093/ee/nvad085 37639676

[CIT0025] Schotzko DJ , Okeeffe LE. 1986. Comparison of sweep net, d-vac, and absolute sampling for *Lygus hesperus* (Heteroptera: Miridiae) in lentils. J. Econ. Entomol. 79:224–228.

[CIT0026] Sivakoff FS , Rosenheim JA, Hagler JR. 2012. Relative dispersal ability of a key agricultural pest and its predators in an annual agroecosystem. Biol. Control 63:296–303. https://doi.org/10.1016/j.biocontrol.2012.09.008

[CIT0027] Strong FE , Sheldahl JA, Hughes PR, et al 1970. Reproductive biology of *Lygus hesperus* Knight. Hilgardia 40:133–147. https://doi.org/10.3733/hilg.v40n04p133

[CIT0028] Swezey SL , Nieto DJ, Hagler JR, et al 2013. Dispersion, distribution, and movement of *Lygus* spp. (Hemiptera: Miridae) in trap-cropped organic strawberries. Environ. Entomol. 42:770–778. https://doi.org/10.1603/EN12353 23905741

[CIT0029] Villavaso EJ. 2005. A non-sticky trap for tarnished plant bug (Heteroptera: Miridae). J. Entomol. Sci. 40:136–142. https://doi.org/10.18474/0749-8004-40.2.136

[CIT0030] Wardle AR , Borden JH, Pierce HD Jr, et al 2003. Volatile compounds released by disturbed and calm adults of the tarnished plant bug, *Lygus lineolaris*. J. Chem. Ecol. 29:931–944. https://doi.org/10.1023/a:1022987901330 12775153

[CIT0031] Williams L , Blackmer JL, Rodriguez-Saona C, et al 2010. Plant volatiles influence electrophysiological and behavioral responses of *Lygus hesperus*. J. Chem. Ecol. 36:467–478. https://doi.org/10.1007/s10886-010-9778-2 20401755

[CIT0032] Witzgall P , Kirsch P, Cork A. 2010. Sex pheromones and their impact on pest management. J. Chem. Ecol. 36:80–100. https://doi.org/10.1007/s10886-009-9737-y 20108027

[CIT0033] Yang CY , Kim S-J, Kang T-J, et al 2015. Sex pheromones and reproductive isolation in five mirid species. PLoS One 10:e0127051. https://doi.org/10.1371/journal.pone.0127051 25973902 PMC4431809

[CIT0034] Zalom FG , Pickel C, Walsh DB, et al 1993. Sampling for *Lygus hesperus* (Hemiptera: Miridae) in strawberries. J. Econ. Entomol. 86:1191–1195. https://doi.org/10.1093/jee/86.4.1191

[CIT0035] Zalom FG , Bolda MP, Dara SK, Joseph S. 2014. Insects and mites. UC IPM pest management guidelines - strawberry. Davis (CA): University of California ANR Publication 3468.

[CIT0036] Zapponi L , Nieri R, Zaffaroni‑Caorsi V, et al 2023. Vibrational calling signals improve the efficacy of pheromone traps to capture the brown marmorated stink bug. J. Pest Sci. 96:587–597. https://doi.org/10.1007/s10340-022-01533-0

[CIT0037] Zhang T , Mei XD, Zhang XF, et al 2020. Identification and field evaluation of the sex pheromone of *Apolygus lucorum* (Hemiptera: Miridae) in China. Pest Manag. Sci. 76:1847–1855. https://doi.org/10.1002/ps.5714 31825553

[CIT0038] Zhang T , Zhang X, Wyckhuys KAG, et al 2021. Optimization and field demonstration of the *Lygus pratensis* (Hemiptera: Miridae) sex pheromone. Pest Manag. Sci. 77:817–823. https://doi.org/10.1002/ps.6083 32926583

