# Supplementary material for: Development of practical pheromone lures for Lygus hesperus and Lygus elisus (Heteroptera: Miridae)
Source: J Econ Entomol. 2024 Nov 22;118(1):184–94. doi: 10.1093/jee/toae266 (PMC11818382; doi:10.1093/jee/toae266)
Supplement: toae266_suppl_Supplementary_Material [file toae266_suppl_supplementary_material.docx]

# Supplementary Materials

# Development of practical pheromone lures for *Lygus hesperus* and *Lygus elisus* (Heteroptera: Miridae)

## David R. Hall^1*^, Jacqueline Serrano^2†^, Glenn Y. Yokota^3^, Diego J. Nieto^4^, Dudley I. Farman^1^, J. Steven McElfresh^2^, Alejandro I. Del Pozo-Valdivia^5††^, Jocelyn G. Millar^2^, Kent M. Daane^3^

#### ^1^ Natural Resources Institute, University of Greenwich, Central Avenue, Chatham Maritime, Kent ME4 4TB, UK. [D.R.Hall@greenwich.ac.uk](mailto:D.R.Hall@greenwich.ac.uk) Orcid 0000-0002-7887-466X.

#### ^2^ Departments of Entomology and Chemistry, University of California, Riverside CA 92521, USA. Serrano ([jacqueline.serrano@usda.gov](mailto:jacqueline.serrano@usda.gov)) Orcid: 0000-0001-8034-6391; McElfresh ([steve.mcelfresh@ucr.edu](mailto:steve.mcelfresh@ucr.edu)) Orcid 0000-0002-8736-6632; Millar ([millar@ucr.edu](mailto:millar@ucr.edu)) Orcid: 0000-0001-7639-9001.

#### ^3^ Department of Environmental Science, Policy and Management, University of California, Berkeley, CA 94720, USA. Yokota ([gyyokota@gmail.com](mailto:gyyokota@gmail.com)); Daane (kdaane@ucanr.edu) Orcid: 0000-0001-8052-1954.

#### ^4^ Entomology Department, Driscoll’s Inc., Watsonville, CA, 95076, USA. [diego.nieto@driscolls.com](mailto:diego.nieto@driscolls.com) Orcid: 0009-0002-1797-6686.

#### ^5^University of California Cooperative Extension, Monterey County, Salinas, CA 93901, USA. [adelpozo@vt.edu](mailto:adelpozo@vt.edu) Orcid: 0000-0001-5327-154X.

^†^Current address: USDA-ARS Temperate Tree Fruit and Vegetable Research Unit, Wapato, WA 98951, USA

^††^Current address: Department of Entomology, Virginia Polytechnic Institute and State University, Virginia Beach, VA 23455, USA.

#### **Fig. S1.** Pheromone trap showing (a) positioning in alfalfa field, and (b) detail showing pipette tip dispenser with blue cap.

#### **Fig. S2.** GC-FID Analysis of volatiles from virgin female *Lygus hesperus* on polar GC column ((*E*)-2-hexenal 5.28 min; 1-hexanol 7.29 min; hexyl butyrate 8.25 min; (*E*)-2-hexenyl butyrate 9.04 min; (*E*)-4-oxo-2-hexenal 10.5 min)

#### **Fig. S3.** Release of hexyl butyrate from single virgin female *Lygus hesperus,* from 10 individual insects over successive light and dark periods

#### **Fig. S4.** Representative analysis of volatiles collected from virgin female *Lygus hesperus* by gas chromatography coupled to electroantennographic recording (GC-EAG) using the antenna of a male bug. Upper trace is EAG signal, lower trace is gas chromatogram. EAG responses (*) to HB, E2HB and E4OH but not to 1-hexanol at 8.08 min.

#### **Fig. S5.** Release rates of hexyl butyrate (HB) and (*E*)-4-oxo-2-hexenal (E4OH) at 27 °C from a polyethylene bulb dispenser loaded with 10 mg HB and 2 mg E4OH, measured by collection of volatiles; results are the mean of two replicates

#### **Fig. S6.** Catches of *Lygus* spp. in traps baited with blends of hexyl butyrate (HB), (*E*)-2-hexenyl butyrate (E2HB) and (*E*)-4-oxo-2-hexenal (E4OH) from literature, based on those reported for *L. lineolaris* by Wardle et al. (2003) (100:27:8), *L. hesperus* by Ho and Millar (2002) (100:4:2) and Byers et al. (2013) (100:4:44), and a blend for *L. rugulipennis* by Fountain et al. (2014) (100:3:20) during 7-26 June and 3-9 July 2012. Means with different letters are significantly different (*P* < 0.05).

#### **Fig. S7.** Catches of *Lygus* spp. in traps baited with (A) blends of hexyl butyrate (HB), (*E*)-2-hexenyl butyrate (E2HB) and (*E*)-4-oxo-2-hexenal (E4OH) during 27 August – 9 September 2013, and (B) blends of E2HB and E4OH during 23 - 30 September and 30 September - 7 October 2013. Means with different letters are significantly different (*P* < 0.05).
